# Microbiome digital signature of MCR genes – an *in silico* approach to study the diversity of methanogenic population in laboratory-developed and pilot-scale anaerobic digesters

**DOI:** 10.1099/acmi.0.000044

**Published:** 2019-07-22

**Authors:** K. Ponni Keerthana, S. Radhesh Krishnan, S. Ragunath Sengali, R. Srinivasan, N. Prabhakaran, G. Balaji, M. Gracy, K. Latha

**Affiliations:** ^1^ R & D Division Extension, T. Stanes and Company Limited, Coimbatore-641018, TN, India

**Keywords:** Anaerobic digester (AD), Biogas, *methyl coenzyme-M reductase α-subunit* (*mcrA*), Cow manure, *Methanocorpusculum* and *Methanosarcinaceae*

## Abstract

The production of biogas by anaerobic digestion (AD) of organic/biological wastes has a firm place in sustainable energy production. A simple and cost-effective anaerobic jar at a laboratory scale is a prerequisite to study the microbial community involved in biomass conversion and releasing of methane gas. In this study, a simulation was carried out using a laboratory-modified anaerobic-jar-converted digester (AD1) with that of a commercial/pilot-scale anaerobic digester (AD2). Taxonomic profiling of biogas-producing communities by means of high-throughput methyl coenzyme-M reductase α-subunit (mcrA) gene amplicon sequencing provided high-resolution insights into bacterial and archaeal structures of AD assemblages and their linkages to fed substrates and process parameters. Commonly, the bacterial phyla *Euryarchaeota *, *Chordata*, *Firmicutes* and *Proteobacteria* appeared to dominate biogas communities in varying abundances depending on the apparent process conditions. Key micro-organisms identified from AD were *Methanocorpusculum labreanum* and *Methanobacterium formicicum *. Specific biogas production was found to be significantly correlating to *Methanosarcinaceae *. It can be implied from this study that the metagenomic sequencing data was able to dissect the microbial community structure in the digesters. The data gathered indicates that the anaerobic-jar system could throw light on the population dynamics of the methanogens at laboratory scale and its effectiveness at large-scale production of bio-methane. The genome sequence information of non-cultivable biogas community members, metagenome sequencing including assembly and binning strategies will be highly valuable in determining the efficacy of an anaerobic digester.

## Introduction

Anaerobic digestion (AD) is a biological treatment performed in the absence of oxygen to stabilize organic matter while producing biogas, a mixture formed mainly of methane and carbon dioxide. The oldest and more widespread application of AD is the treatment of sewage sludge (SS) and it experienced an important growth after the first energy crisis in the 1970s, especially with the appearance of immobilized biomass systems to treat soluble effluents, and now considered as a developed technology [1]. Anaerobic digestion (AD) of organic matter is widely used in the treatment of agricultural and food wastes as well as industrial and domestic wastewaters. Moreover, the production of biogas from renewable biomass is a substantial way to partially shift from fossil fuels to renewable greenhouse gas-neutral bioenergy in order to mitigate the climate change. Currently, AD is intensively applied for the generation of clean energy and high-quality organic fertilizers from various organic substrates in many countries [2–5].

Ruminants have evolved an efficient digestive system in which microbes ferment the plant material that constitutes the animal’s diet to produce short chain fatty acids, principally acetic, propionic and butyric acids, and other products [6, 7]. This fermentation is carried out by a complex microbial community, which includes bacteria, ciliate protozoa, anaerobic fungi and methanogenic archaea, and has been the focus of numerous studies. The role of the methanogenic archaea in the rumen environment is important as they use hydrogen (H_2_) derived from microbial fermentation as their energy source and combine it with carbon dioxide (CO_2_) to form methane (CH_4_), which is eructed from the animal and released to the atmosphere. Other fermentation end-products, including formate and methyl-containing compounds, can also be substrates for methanogenesis [8].

Methane is a potent greenhouse gas contributing to global climate change, and ruminant-derived CH_4_ accounts for about one-quarter of all anthropogenic CH_4_ emissions [9]. Development of strategies to reduce CH_4_ emissions from farmed animals are currently being investigated, and methanogen-genome-sequence information has already been used to inform CH_4_-mitigation strategies based on vaccines and small-molecule inhibitors [8, 10]. CH_4_-mitigation technologies should target features that are conserved across all rumen methanogens, and be methanogen-specific so that other rumen microbes can continue their normal digestive functions. The syntrophic relationship between bacteria oxidizing organic acids and alcohols and methanogenic archaea is essential for the AD process. The chief enzyme in this pathway, the *mcrA*, catalysis the final step in methanogenesis and the initial step in methanotrophy [11].

Thus, the composition and dynamics of the methanogenic communities were investigated in laboratory-scale AD targeting the *mcrA* genes in the present study. Furthermore, physiochemical characterization of the inoculum, digested/fermented sludge and biogas potential of the laboratory-scale digester and the pilot-scale digester were analysed to validate the potential of the microbes identified in both the digesters.

## Methods

### Laboratory-scale anaerobic digester

**Fig. 1.**
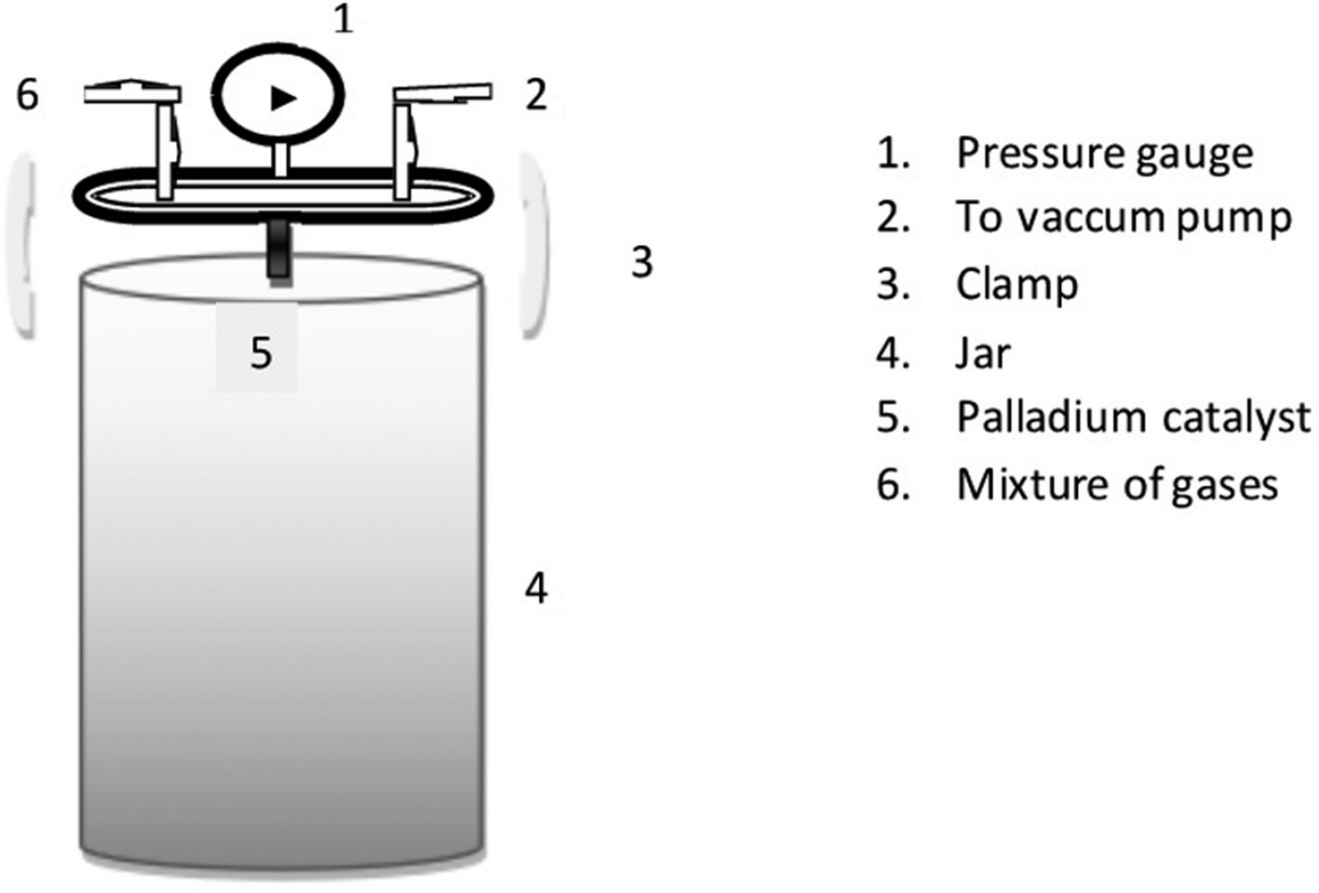
Outline representation of the experimental jar.

An experimental jar made of polycarbonate with an external pressure gauge was used for anaerobic conditions (Fig. 1). The pressure gauge indicates the gas produced and is measured in p.s.i. (pound per square inch) and kg cm^−^^2^. The value on the gauge ranges from −14.69 to 30 p.s.i. equivalent to 2 kg cm^−^^2^. The total capacity of the jar is 2.5 L, with a working capacity of 2 l. The fermented waste of bio-production unit from T. Stanes and Company Limited was used as substrate for biogas generation.

### Standardization of inoculum for biogas generation

#### Prime inoculum

**Fig. 2.**
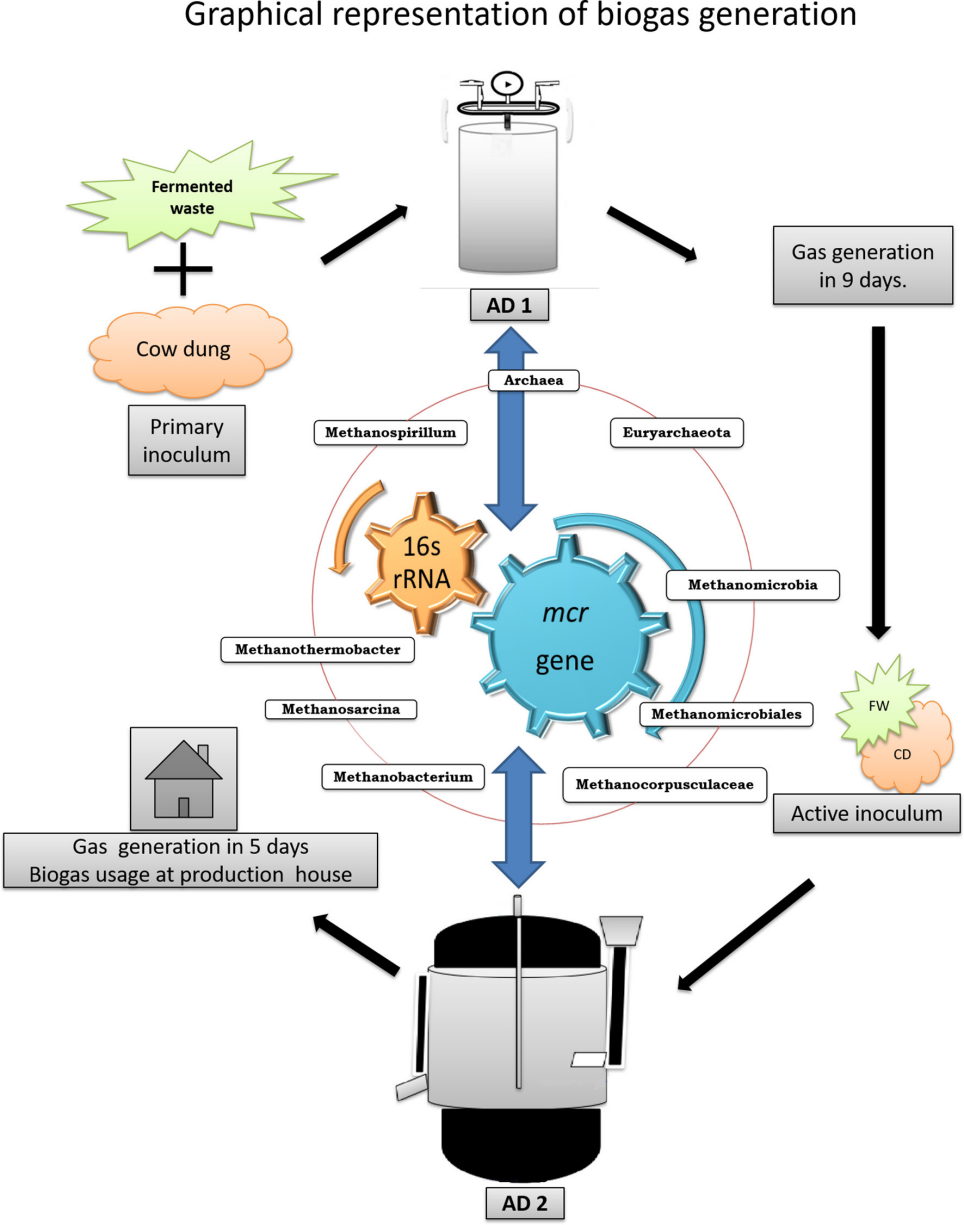
Standardization of active inoculum from fermented waste for biogas generation. Biogas generation by anaerobic digestion and microbiome Digital signature of MCR genes to study the diversity of methanogenic population in laboratory-developed and pilot-scale anaerobic digesters.

The seed inoculum was prepared from the starter inoculum (cow manure), by conditioning, the microbes with periodic addition of fermented waste for effective degradation of the fermented waste and followed by biogas generation. The experimental jar was set up with cow manure, fermented waste and water in with 10 % primary inoculum under vacuum, and the resultant inoculum served as the secondary inoculum after incubation at room temperature for 5–7 days and as prime inoculum for further studies. The pH was noted at the end of the incubation, with periodic monitoring of gas by flame test and portable gas analyser. Inoculum was found to generate a high amount of methane and comparable to the commonly generated biogas and served as the mother inoculum for pilot-scale generation of biogas. The prime mother inoculum was preserved at 4 °C, for isolation, identification and characterization of the potential micro-flora by metagenomics. The overall inoculum standardization depicted in (Fig. 2).

#### Pilot-scale biogas generation with fermented waste

**Fig. 3.**
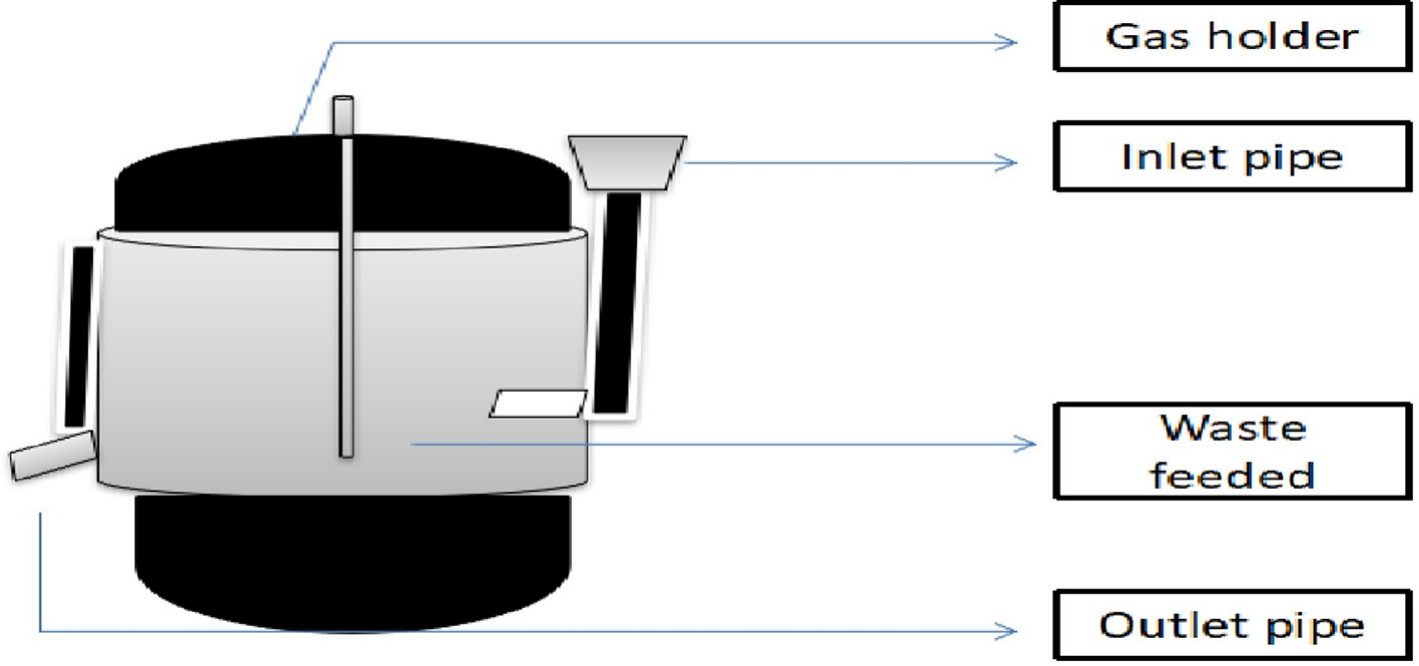
Outline representation of the pilot-scale biogas digester.

The experimental trial was conducted in a semi-pilot-scale bio-methanation/AD2 plant. The capacity of the AD2 plant (500L) was used for biogas generation from fermented waste (Fig. 3). Initially the prime inoculum was added as the mother inoculum for the biogas generation with repeated addition of fermented waste obtained from the production unit of T. Stanes and Company Limited, Coimbatore India. The periodic addition of fermented waste leads to gas generation, which was tested with a flame test and analysed in a potable gas analyser.

### Analytical methods

#### Physicochemical analysis

The substrate used for generation of bio-methane subjected to physiochemical analysis during, before and after digestion. The parameters include pH, moisture, total solids and volatile solids.

#### Performance assessment of anaerobic digesters for biogas production

The following parameters used for assessing the performance of the digester that includes gas production time, peak production, daily gas yield, flammability test over the hydraulic retention time (HRT) of the study [12]. The combustibility of the biogas produced was determined by flame test and apart from these, the gas was analysed for combustible gas (LEL), carbon dioxide (CO_2_), hydrogen sulfide (H_2_S) and carbon monoxide (CO) using a portable gas analyser/detector.

### DNA extraction and PCR amplification 16s rRNA gene and *mcrA* gene

DNA extraction and purification was performed using the following method. Approximately 700 µl of the sample along with equal volume of the phenol:chloroform:isoamyl alcohol (PCI) (25 : 24 : 1) was added along with four glass beads. The mixture was vortexed vigorously for 2 min and kept in ice for 10 min. Samples were centrifuged at 10 000 ***g*** for 10 min at 4 ˚C. Aqueous phase was mixed with equal volume of PCI (25 : 24 : 1) and the same protocol as mentioned above repeated. Aqueous-phase ice-cold isopropanol was added and incubated at −20 ˚C for 1 h. The mixture was centrifuged for 10 min for 10 000 ***g*** at 4 ˚C and the pellet was washed with 70 % ethanol. The air-dried pellets were diluted the sterile milli-Q water. DNA was checked for integrity by agarose gel electrophoresis and quantified with a NanoDrop ND-1000 UV-Vis spectrophotometer (Thermo Fisher Scientific).

PCR amplifications were performed with a Bio-Rad thermal cycler (Bio-Rad, Germany). The PCR mix consisted of 1× PCR buffer, a 0.2 µM concentration (each) of forward and reverse primers, a 2.5 mM concentration of each deoxynucleoside triphosphate (dNTP), 1U Taq DNA polymerase and 30 ng of template DNA.

For amplification of 16S rRNA, the following specific primers were used: 27F (5′ AGAGTTTGATCMTGGCTCAG-3′ and 1492R (5′-TACGGYTACCTTGTTACGACTT-3′) and for the amplification *mcrA* regions, the following specific degenerate [11] primers were used: MCR FP (5′ GGTGGTGTMGGATTCACACARTAYGCWACAGC-3′) and MCR RP (5′- TTCATTGCRTAGTTWGGRTAGTT-3′). The PCR protocol used included an initial DNA denaturation for 2.0 min at 95 °C; 25 cycles of 95 °C for 30 s, annealing at 60 °C for 30 s, and elongation at 72 °C for 2 min; 10 min at 72 °C (final extension); and incubation at 4 °C until samples were processed further.

### Purification of PCR production

Removed unincorporated PCR primers and dNTPs from PCR products by using Montage PCR Clean up kit (Thermo Fisher Scientific). The quality, quantity (App. 100 ng µl^−^^1^) and formulation of the PCR product was checked using Qubit Fluorometer 3.0.

### Sequencing protocol

Nanopore sequencing was performed by using 1 µg of DNA as a template. The process was initiated by an End repair/dA tailing, Ligation of Barcode Adapter, Barcoding PCR, End repair/dA tailing, Blunt end Adapter Ligation, Purification using AMPure XP bead binding, Priming and loading the SpotON flow cell.

### Bioinformatics protocol

EPI2ME 16S analysis workflow allows users to perform genus-level identification from single reads; with access to basecalled files for detailed investigations at the species and sub-species level. The phylogeny analysis of query sequence with the closely related sequence of blast results that was performed followed by multiple sequence alignment. The workflow is designed to blast basecalled sequence against the NCBI MCR region bacterial database, which contains MCR sequences from different organisms. Each read is classified based on % coverage and identity. The MCR workflow will be useful in identifying pathogens in a mixed sample or understanding the composition of a microbial community.

## Results

### Methane-rich inoculum

**Fig. 4.**
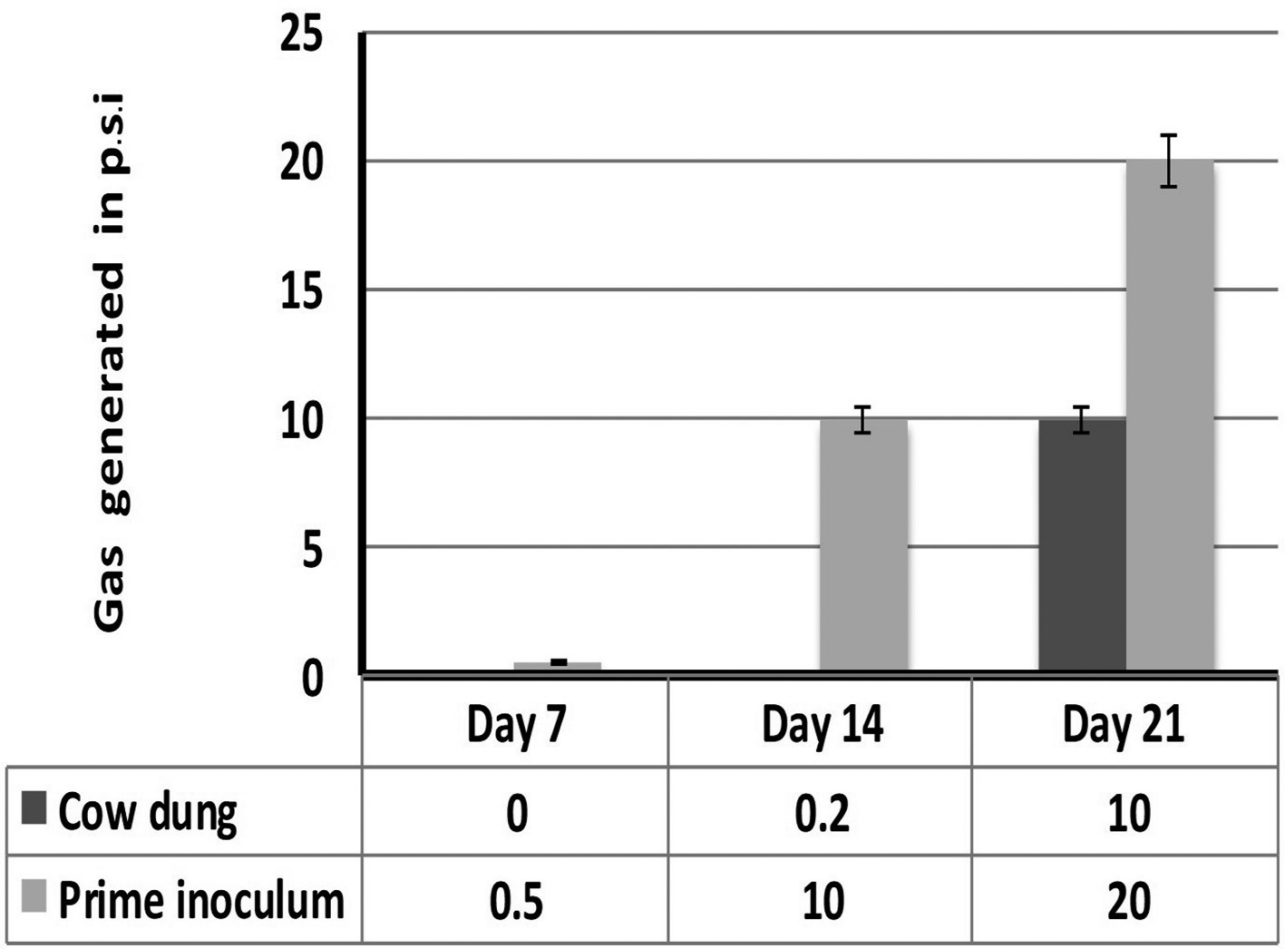
Biogas generation of inoculum.

The inoculum showed a profound increase in methane generation compared to regular biogas generation from cow manure. The optimum HRT was observed to be 21 days (Fig. 4).

**Fig. 5.**
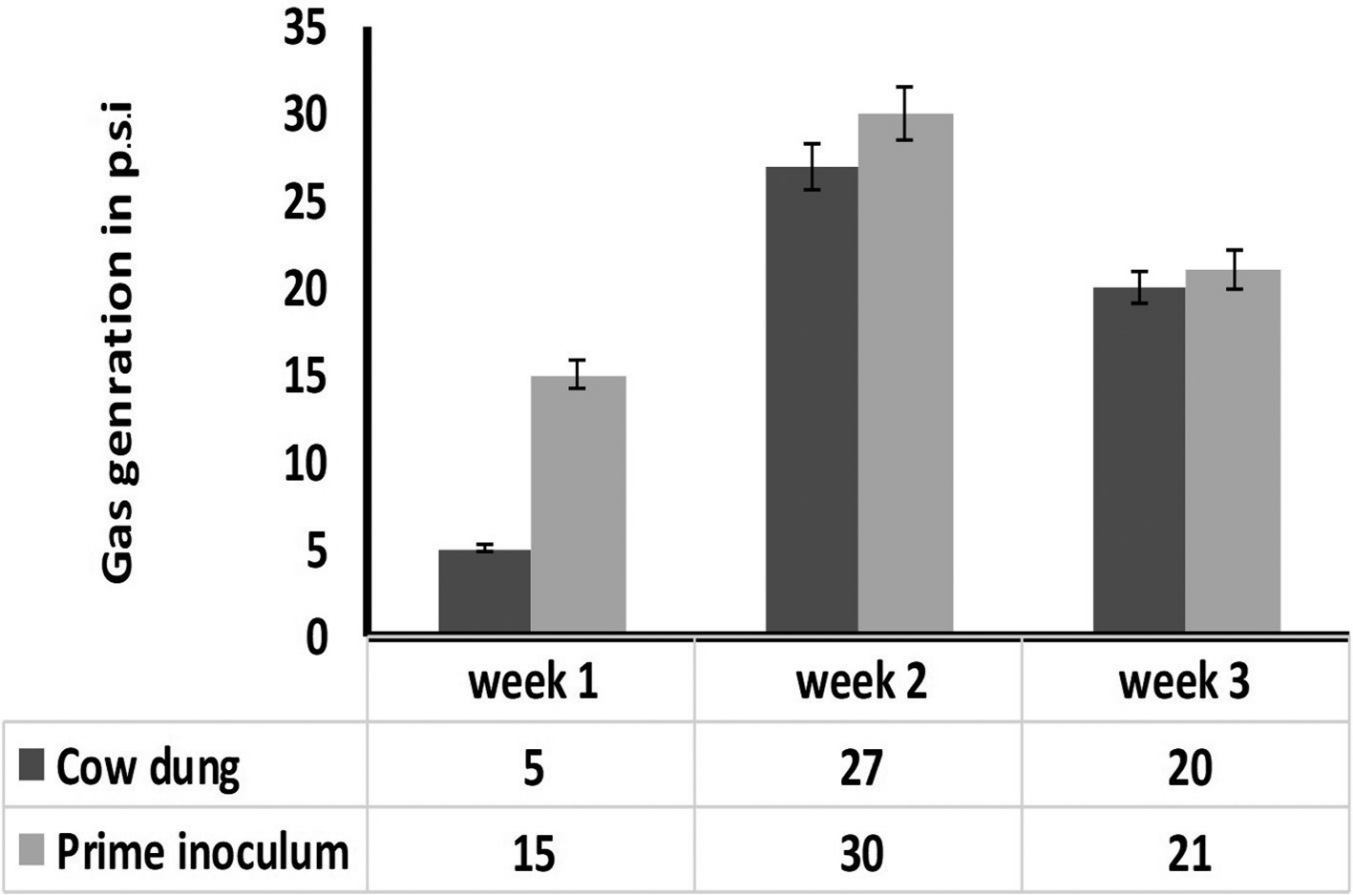
Volume of biogas produced against retention time in cow dung and prime Inoculum.

The pH of the reactor increased to alkaline indicating the degradation of the production waste by the micro-organisms (Table 1), where the reduction in the total solids and volatile solids have been reported by [13] due to the utilization of the substrate by micro-organisms, which in turn increases the methane yield. A higher biogas generation was observed in the prime inoculum than the cow manure consistently for 3 weeks (Fig. 5).

**Table 1.** Physiochemical parameters of the substrate

**S**. **no**.	**Parameters**	**Fermented waste before digestion**	**Fermented waste after digestion**
1.	pH	7.00	8.82
2.	Moisture %	99	98.57
3.	Total solids %	2.34	0.95
4.	Volatile residue %	1.25	0.68

The solid retention time equals to the HRT of the digester. The retention time plays a significant role in the methane generation of the digester. During first week gas production was low and was slowed, with peak production in the second week followed by a slow decline in third week indicating that the retention time of the substrate is 3 weeks and is comparable to the regular biogas generated from cow manure [14].

### Performance and flammability assessment between AD1 and AD2

**Fig. 6.**
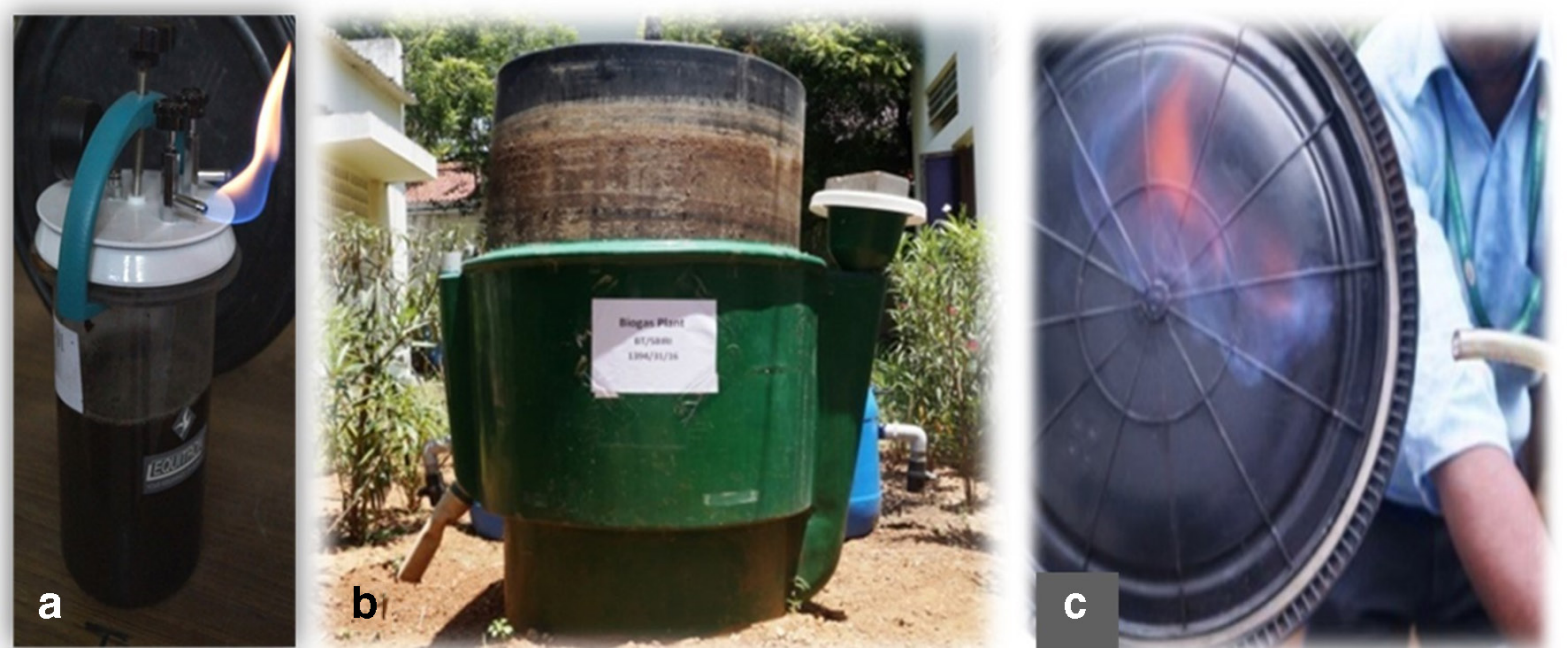
Flame test with the methanation plants. (a) Lab-scale bench-top methanation unit. (b) Pilot-scale bio-methanation plant and (c) flame test.

The initial and final pH ranged from 6 to 7 is optimum for growth of methanogens. The peak production with respect to HRT was observed to be higher in AD1 followed by AD2 (Table 2). The flammability of the gas produced from the production was equally efficient in both digesters (lab and pilot) but more rapid than the conventional generation of biogas (Fig. 6).

**Table 2.** Performance assessment between AD1 and AD2 in comparison with cow manure

	**Parameters**	**Cow manure**	**AD1**	**AD2**
1.	Initial pH	6.71	7.00	7.30
2.	Biogas production started	Second day	Second day	Second day
3.	Flammability produced	Seventh day	Fourth day	Fifth day
4.	Flammability test	+++	+++	+++
5.	Peak production	Twentieth day	Ninth day	Fifth day
6.	Production stopped	Twentieth day	Twenty-first day	−−
7.	Daily gas produced in m^3^	0.15	0.2	1
8.	Final pH	7.10	7.15	7.41

+++,Highly flammable.

−−, Continuous generation with no decrease in production.

The gas analysed for combustibility showed 99–100 % after with trace amount of hydrogen sulphide and carbon monoxide (Table 3). The biogas production rate was consistent and maintained due to the continued growth of methanogens.

**Table 3.** Biogas composition check using a portable gas analyser

**S**. **no**.	**Gas name**	**AD1**	**AD2**
**1**.	Combustible gas (LEL)	100 %	100 %
**2**.	Carbon di oxide (CO_2_)	0 p.p.m.	0 p.p.m.
**3**.	Hydrogen sulphide (H_2_s)	98 p.p.m.	100 p.p.m.
**4**.	Carbon monoxide (Co)	1.3 %	0.5 %

### Methanogenic community composition based on *mcrA* gene analysis

Methanogenic archaeal community dynamics in AD (1 and 2) were tracked by *mcrA* gene analysis from metagenome. A total of 368 and 380 cumulative reads were predicted from the AD1 and AD2 metagenomes, respectively. The majority of *mcrA* sequences were closely related to clones from various anaerobic digesters. In general, a good agreement was obtained with *mcrA* analyses. No significant differences were observed in the community structure of the AD system.

From AD1 alone a total of 323 cumulative reads were predicted to be *Methanomicrobia* and *Methanobacteria* (Tables 4 and 5). Predictions from families like *Methanocorpusculaceae *, *Methanomicrobiaceae *, *Methanobacteriaceae *, *Methanosarcinaceae* and *Methanoregulaceae* were found to be dominant in AD1. Organisms from other families like *Xylonomycetaceae*, *Leptosphaeriaceae*, *Glomerellaceae*, *Porphyromonadaceae *, *Pasteurellaceae *, *Alteromonadaceae *, *Rhodobacteraceae *, *Dehalococcoidaceae *, *Bacillaceae *, *Paenibacillaceae *, *Staphylococcaceae *, *Leuconostocaceae *, *Lactobacillaceae *, *Clostridiaceae* and *Oscillospiraceae* were also predicted from the AD1 metagenome. The data generated was submitted to NCBI (accession: PRJNA551113 ID: 551113).

**Table 4.** MCR report AD1 sequence analysis from metagenomic DNA

**Super Kingdom**		**Phylum**		**Class**		**Order**		**Family**		**Genus**		**Species**	
Archaea	323	Euryarchaeota	323	Methanomicrobia	239	Methanomicrobiales	215	* Methanocorpusculaceae *	116	* Methanocorpusculum *	116	* Methanocorpusculum labreanum *	116
Bacteria	24	Firmicutes	12	Methanobacteria	84	Methanobacteriales	84	* Methanomicrobiaceae *	97	* Methanoculleus *	97	* Methanobacterium formicicum *	55
Eukaryota	21	Chordata	11	Mammalia	11	Methanosarcinales	24	* Methanobacteriaceae *	84	* Methanobacterium *	83	* Methanoculleus marisnigri *	50
**Super Kingdom**		Ascomycota	10	Bacilli	7	Primates	11	* Methanosarcinaceae *	23	* Methanosarcina *	23	* Methanobacterium* sp. *MB1*	24
Proteobacteria	6	Gammaproteobacteria	5	Lactobacillales	4	*Hominidae*	11	*Homo*	11	* Methanoculleus bourgensis *	16
Bacteroidetes	3	Eurotiomycetes	4	Eurotiales	3	*Aspergillaceae*	3	*Aspergillus*	3	* Methanosarcina barkeri *	12
Chloroflexi	1	Bacteroidia	3	Bacteroidales	3	* Enterobacteriaceae *	3	* Veillonella *	3	*Homo sapiens*	11
		**Phylum**		Negativicutes	3	Enterobacterales	3	* Veillonellaceae *	3	*Metschnikowia*	2	* Methanosarcina mazei *	8
Saccharomycetes	2	Bacillales	3	*Metschnikowiaceae*	2	* Prevotella *	2	* Methanoculleus* sp. *MAB1*	2
Clostridia	2	Veillonellales	3	* Prevotellaceae *	2	* Streptococcus *	2	*Metschnikowia bicuspidata*	2
Xylonomycetes	1	Saccharomycetales	2	* Streptococcaceae *	2	* Methanothermobacter *	1	*Plautia stali symbiont*	2
Dothideomycetes	1	Clostridiales	2	* Methanoregulaceae *	1	* Methanoregula *	1	* Veillonella parvula *	2
Sordariomycetes	1	Onygenales	1	* Methanospirillaceae *	1	* Methanospirillum *	1	* Methanoregula formicica *	1
Alphaproteobacteria	1	Xylonomycetales	1	* Methanosaetaceae *	1	* Methanothrix *	1	* Methanospirillum hungatei *	1
Dehalococcoidia	1	Pleosporales	1	*Xylonomycetaceae*	1	*Coccidioides*	1	* Methanothrix soehngenii *	1
				**Class**		Glomerellales	1	*Leptosphaeriaceae*	1	*Xylona*	1	*Coccidioides immitis*	1
Pasteurellales	1	*Glomerellaceae*	1	*Leptosphaeria*	1	*Xylona heveae*	1
Alteromonadales	1	* Porphyromonadaceae *	1	*Colletotrichum*	1	*Leptosphaeria maculans*	1
Rhodobacterales	1	* Pasteurellaceae *	1	* Porphyromonas *	1	*Colletotrichum orchidophilum*	1
Dehalococcoidales	1	* Alteromonadaceae *	1	* Salmonella *	1	* Porphyromonas gingivalis *	1
						**Order**		* Rhodobacteraceae *	1	* Haemophilus *	1	* Prevotella intermedia *	1
* Dehalococcoidaceae *	1	* Alteromonas *	1	* Salmonella enterica *	1
* Bacillaceae *	1	* Ketogulonicigenium *	1	* Haemophilus parainfluenzae *	1
* Paenibacillaceae *	1	* Dehalococcoides *	1	* Dehalococcoides mccartyi *	1
* Staphylococcaceae *	1	* Virgibacillus *	1	* Virgibacillus halodenitrificans *	1
* Leuconostocaceae *	1	* Paenibacillus *	1	* Paenibacillus yonginensis *	1
* Lactobacillaceae *	1	* Staphylococcus *	1	* Staphylococcus epidermidis *	1
* Clostridiaceae *	1	* Weissella *	1	* Weissella cibaria *	1
* Oscillospiraceae *	1	* Lactobacillus *	1	* Clostridium argentinense *	1
**Family**		* Clostridium *	1	* Oscillibacter valericigenes *	1
* Oscillibacter *	1	* Veillonella rodentium *	1
Total	368		366		365		365		364		363		319

**Table 5.** MCR region AD2 sequence analysis from Metagenomic DNA

**Super kingdom**	**Phylum**	**Class**	**Order**	**Family**	**Methanobacterium**	**Species**	
Archaea	373	Euryarchaeota	373	Methanobacteria	202	Methanobacteriales	202	* Methanobacteriaceae *	201	* Methanobacterium *	197	* Methanobacterium formicicum *	185
Eukaryota	4	Chordata	4	Methanomicrobia	142	Methanomicrobiales	123	* Methanoregulaceae *	54	* Methanoregula *	54	* Methanoregula formicica *	29
Bacteria	3	Firmicutes	2	Methanococci	18	Methanococcales	18	* Methanocorpusculaceae *	27	* Methanocorpusculum *	27	* Methanocorpusculum labreanum *	27
**Super Kingdom**		Proteobacteria	1	Mammalia	4	Methanosarcinales	17	* Methanosarcinaceae *	17	* Methanosarcina *	16	* Methanoregula boonei *	19
	**Phylum**		Bacilli	2	Primates	4	* Methanococcaceae *	14	* Methanococcus *	14	* Methanococcus maripaludis *	14
		Thermoplasmata	1	Bacillales	2	* Methanomicrobiaceae *	12	* Methanospirillum *	12	* Methanospirillum hungatei *	12
		Gammaproteobacteria	1	Methanomassiliicoccales	1	* Methanospirillaceae *	12	* Methanoculleus *	7	* Methanosarcina mazei *	8
		**Class**		Enterobacterales	1	* Methanocaldococcaceae *	4	* Methanolacinia *	5	* Methanolacinia petrolearia *	5
			**Order**		*Hominidae*	4	* Methanothermobacter *	4	* Methanotorris igneus *	4
				* Methanothermaceae *	1	* Methanotorris *	4	*Homo sapiens*	4
				* Methanomassiliicoccaceae *	1	*Homo*	4	* Methanobacterium* sp. *MB1*	3
				* Enterobacteriaceae *	1	* Methanothermus *	1	* Methanothermobacter marburgensis *	2
				* Bacillaceae *	1	* Methanolobus *	1	* Methanoculleus marisnigri *	2
				* Planococcaceae *	1	* Methanomassiliicoccus *	1	* Methanobacterium paludis *	1
				**Family**		* Bacillus *	1	* Methanothermus fervidus *	1
					* Solibacillus *	1	* Methanoculleus bourgensis *	1
					**Methanobacterium**		* Methanosarcina barkeri *	1
						* Methanosarcina lacustris *	1
						*Methanolobus psychrophilus*	1
						*Candidatus methanomassiliicoccus intestinalis*	1
						* Bacillus subtilis *	1
						* Solibacillus silvestris *	1
						*Plautia stali symbiont*	1
Total predictions	380		380		370		368		350		349		324

## Correlations between methanogenic communities

A similar pattern of predictions was observed from AD2. A total of 380 predictions belonging to superkingdoms like Archaea, Eukaryota and Bacteria were derived from the AD2 metagenome. Of these, methanobacterium alone constituted 349 reads. Methanogenic community based on the *mcrA* gene profiles in AD1 was dominated by members of the hydrogenotrophic *Methanoculleus* genus.

**Fig. 7.**
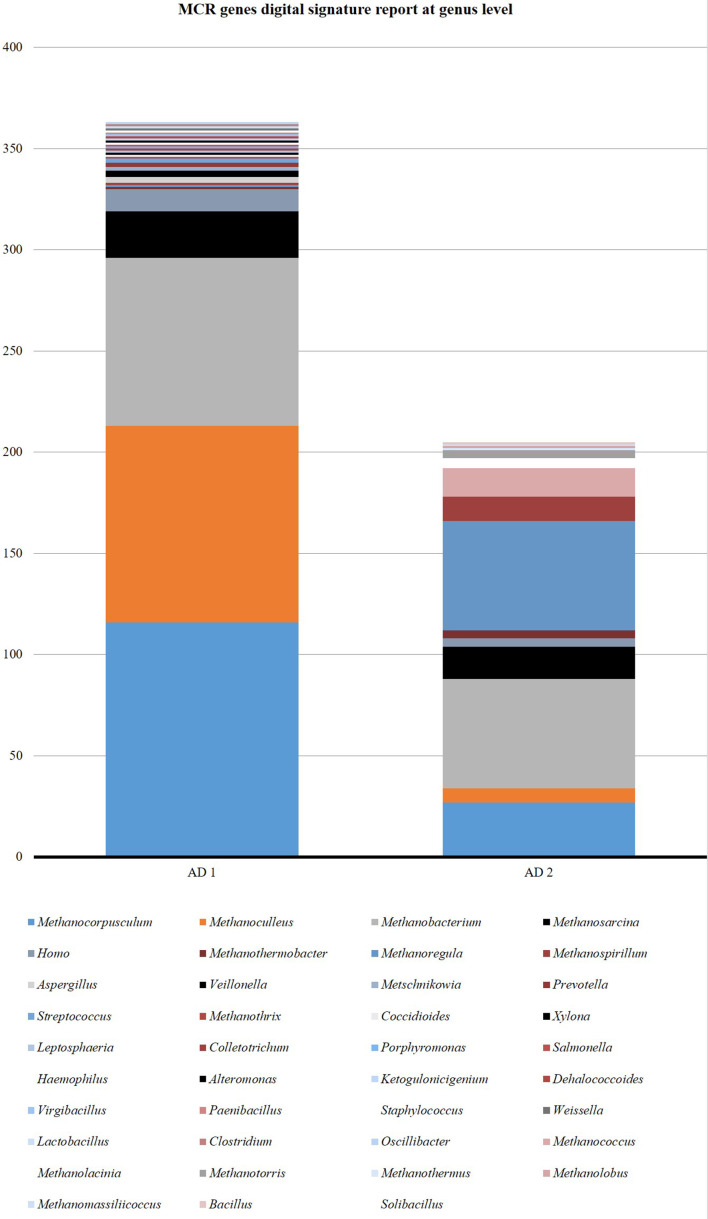
Comparison between two anaerobic digesters at genus level

**Fig. 8.**
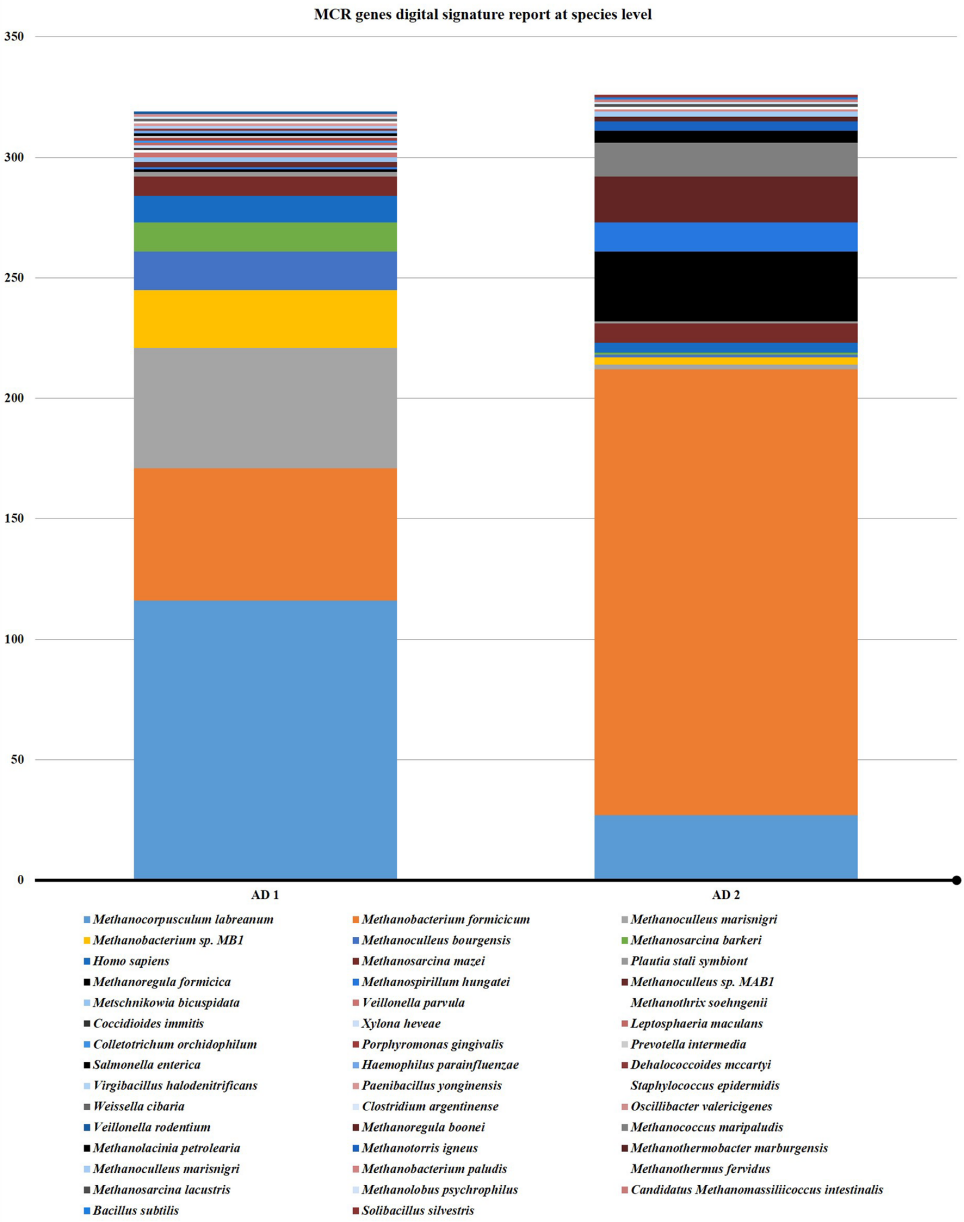
Comparison between two anaerobic digesters at species level.

Organisms like *Methanocorpusculum labreanum, Methanobacterium formicicum, Methanoculleus marisnigri, Methanobacterium* sp*. MB1, Methanoculleus bourgensis, Methanosarcina barkeri, Homo sapiens, Methanosarcina mazei, Plautia stali symbiont, Methanoregula formicica* and *Methanospirillum hungatei* were common in both the anaerobic digesters (Figs 7 and 8)

(For more details please see Tables S1 and S2, available in the online version of this article).

A higher cumulative read of *Methanocorpusculum *, *Methanoculleus *, *Methanobacterium* and *Methanosarcina* was observed from AD1 than AD2. Species like *Methanocorpusculum labreanum, Methanobacterium formicicum, Methanoculleus marisnigri, Methanobacterium* sp. *MB1, Methanoculleus bourgensis, Methanosarcina barkeri* and *Methanosarcina mazei* were dominant in both the anaerobic digester (Table 6).

**Table 6.** Common methanogen found in AD1 and AD2 predicted and its importance

**Species**	**Description**
* Methanocorpusculum labreanum *	Belonging to the order *Methanomicrobiales* within the archaeal kingdom Euryarchaeota [15]
* Methanobacterium formicicum *	Production of methane from formate and H_2_/CO_2_, but not from acetate, alcohols or methylamines. Temperature range (25–45 °C) and pH 6–8. [10]
* Methanoculleus marisnigri *	Isolated from sediment from the Black Sea and freshwater sediments. They use hydrogen, formate and secondary alcohols, such as propanol and butanol, for methanogenesis.
* Methanobacterium* sp. *MB1*	Hydrogenotrophic methanogenic Archaeon isolated from a rural biogas plant producing methane-rich biogas from maize silage and cattle manure [16, 17].
* Methanoculleus bourgensis *	Hydrogenotrophic partner of mesophilic acetate-oxidizing bacteria, a syntrophic relationship operating close to the thermodynamic equilibrium and in ammonia-rich engineered biogas processes [18].
* Methanoculleus* sp. *MAB1*
* Methanosarcina barkeri *	These single-celled organisms are known as anaerobic methanogens that produce methane using all three metabolic pathways for methanogenesis
* Methanosarcina mazei *
* Methanospirillum hungatei *	This genus and species name was first proposed in 1974 by Ferry *et al*. [19]. Treatment of waste and in bioenergy industries by breaking down organic wastes and production of methane.

## Discussion

### Efficient inoculum to produce biogas rich in methane

The prime inoculum was developed with co-fermentation of fermented waste with cow manure resulted in stable methane generation. Methanogens are sensitive to both higher and lower pH and occurs optimum between 6.5 and 8 [20] and both the digesters were neutral with no observed acid buffering that allowed the digestion process to enhance the growth of methanogens. A reduction in volatile residue and total solids was observed, which is due to the consumption of waste by the microbial flora. Our findings correlate with earlier reports [13, 21] who specified that, the total solids and volatile solids decrease as methane yield increase. A high methane gas production was noted due to decline in volatile residue during the third week of the anaerobic digestion process. A similar report was illustrated earlier [22] thereby substantiating our findings.

### Rapid biogas generation and high HRT

Among various micro-organisms involved in biogas generation, methanogens are very sensitive to different environmental factors, such as high ammonia, sulfide and organic acid concentrations, leading to process impairments. The common reaction of methanogens was reduction of CO_2_ to CH_4_ with H_2_ as the electron donor [23]. The peak production observed earlier due to stable utilization of the substrate that was validated with gas analysis showed a trace amount of toxic gases like H_2_S and CO. Thus, the substrate and inoculum in the AD resulted in good methane generation. The batch reactors are a simple and cheaper form of digestion where the biogas production occurs in a normal distribution pattern over time. Similarly, the AD1 digester at lab scale can be efficiently used for inoculum development and new substrate experiements for biogas generation with reduced equipment cost, easy monitoring and handling.

### Identification of key players by the taxonomic analysis of the microbial community

Despite the fact that comparable results were obtained using both (16 s rRNA and *mcrA*) approaches, *mcrA* data additionally allowed the identification of various members of the family *Methanobacteriaceae,* which were missed with the applied 16S rRNA (data not shown) gene-specific primer set in the previous study. Moreover, the relative abundance data based on the *mcrA* gene were less biased compared to the rRNA gene-based approach, which is impacted by the gene copy number variability. It also appeared that a high proportion of 16S rRNA sequences can only be classified on higher taxonomic ranks indicating that many community members and their participation in AD within functional networks are still unknown.

Based on the analysis of *mcrA* as well as 16S rRNA genes, the methanogenic community in reactor AD1 was less diverse compared to AD2 and were dominated by members of the genus *Methanoculleus *, indicating strong inhibition of the acetoclastic pathway of methanogenesis. The closely related strains are hydrogenotrophic methanogens that can utilize H_2_/CO_2_ or formate as methanogenic substrates.

The next group, members of that were found at high levels in all biogas reactor systems but in various proportions, was the genus *Methanosarcina *. A cumulative read of 39 was noted from the *mcrA* gene. The related strains are acetoclastic and methylotrophic methanogens and that also utilize H_2_/CO_2_ as methanogenic substrates. In fact, even in the absenteeism of acetotrophic *Methanosaetaceae *, anaerobic acetate oxidation to CO_2_ and H_2_ by syntrophic acetate-oxidizing bacteria can be the major pathway for Methanobacteriales and *Methanomicrobiales* above *Methanosarcinales* [24].

Gowdaman and Srikanth, [25] reported *Methanocorpusculum labreanum* is a dominant species in an anaerobic digester operative on food waste for the first time in anaerobic digester and Lee *et al*. [26] found *Methanobacterium formicicum* were dominant in anaerobic digester combined with steady-state microbial electrolysis cells. Our study revealed that the methanogenic bacteria *Methanocorpusculum labreanum* and *Methanobacterium formicicum* were found in both anaerobic digesters (AD1 and AD2).

Analysis of the molecular inventory of methanogens in a thermophilic fermenter showed an all-time dominance of hydrogenotrophic *Euryarchaeota *, although the inoculum used typically comprises both acetoclastic and hydrogenotrophic methanogens. The dominance of H_2_-oxidizing *Methanobacteriales* organisms might be due to their higher specific growth rate than that of acetate-utilizing methanogens. Therefore, a detailed understanding of the microbial consortia in biogas reactors is important to fundamentally and practically develop and improve anaerobic digestion processes [27–30].

## Conclusion

Although cultivation-based approaches to isolate micro-organisms from biogas-fermentation samples yields hundreds of novel species and strains, this approach intrinsically is limited to the cultivable fraction of the community. This study investigated the composition and dynamics of methanogenic communities based on *mcrA* genes in laboratory-scale biogas reactors operated with industrial waste materials. The present study shows that application of methanogenic communities in biogas reactors adapted to specific feedstock might improve the anaerobic digestion of such waste materials in full-scale biogas reactors. Furthermore, the relative abundance data obtained by the *mcrA* gene gives better results since the 16S rRNA gene data is more biased due to the different copy numbers of rRNA operons in various archaeal taxa.

## Author contributions

**Conceptualisation**:

Keerthana Ponni K, Radhesh Krishnan S, Sengali Ragunath K, Srinivasan R, Latha K - Ideas; formulation or evolution of overarching research goals and aims.

**Methodology**:

Keerthana Ponni K, Radhesh Krishnan S, Sengali Ragunath K, Srinivasan R, Latha K - Development or design of methodology; creation of models.

**Software**:

Radhesh Krishnan S, Prabhakaran N - Programming, software development; designing computer programs; implementation of the computer code and supporting algorithms; testing of existing code components.

**Validation**:

Keerthana Ponni K, Radhesh Krishnan S - Verification, whether as apart of the activity or separate, of the overallreplication/reproducibility of results/experiments and other research outputs.

**Formal analysis**:

Keerthana Ponni K, Radhesh Krishnan S, Sengali Ragunath K, Srinivasan R, Latha K - Application of statistical, mathematical, computational, or other formal techniques to analyse or synthesise study data.

**Investigation**:

Keerthana Ponni K, Radhesh Krishnan S, Sengali Ragunath K, Srinivasan R, Balaji G, Gracy M, Latha K - Conducting a research andinvestigation process, specifically performing the experiments, ordata/evidence collection.

## Supplementary Data

Supplementary material 1
