# Supplementary material for: Microbiome digital signature of MCR genes – an in silico approach to study the diversity of methanogenic population in laboratory-developed and pilot-scale anaerobic digesters
Source: Access Microbiol. 2019 Jul 22;1(5):e000044. doi: 10.1099/acmi.0.000044 (PMC7470284; doi:10.1099/acmi.0.000044)
Supplement: Supplementary material 1 [file acmi-1-044-s001.pdf]

Supplementary Table 1. MCR genes Digital signature report at Genus level

	Genus	AD 1	AD 2
1	Methanocorpusculum	116	27
2	Methanoculleus	97	7
3	Methanobacterium	83	54
4	Methanosarcina	23	16
5	Homo	11	4
6	Methanothermobacter	1	4
7	Methanoregula	1	54
8	Methanospirillum	1	12
9	Aspergillus	3	
10	Veillonella	3	
11	Metschnikowia	2	
12	Prevotella	2	
13	Streptococcus	2	
14	Methanothrix	1	
15	Coccidioides	1	
16	Xylona	1	
17	Leptosphaeria	1	
18	Colletotrichum	1	
19	Porphyromonas	1	
20	Salmonella	1	
21	Haemophilus	1	
22	Alteromonas	1	
23	Ketogulonicigenium	1	
24	Dehalococcoides	1	
25	Virgibacillus	1	
26	Paenibacillus	1	
27	Staphylococcus	1	
28	Weissella	1	
29	Lactobacillus	1	
30	Clostridium	1	
31	Oscillibacter	1	
32	Methanococcus		14
33	Methanolacinia		5
34	Methanotorris		4
35	Methanothermus		1
36	Methanolobus		1
37	Methanomassiliicoccus		1
38	Bacillus		1
39	Solibacillus		1
		363	206

Supplementary Table 2. MCR genes Digital signature report at Species level

	Species	AD 1	AD 2
1	Methanocorpusculum labreanum	116	27
2	Methanobacterium formicicum	55	185
3	Methanoculleus marisnigri	50	2
4	Methanobacterium sp. MB1	24	3
5	Methanoculleus bourgensis	16	1
6	Methanosarcina barkeri	12	1
7	Homo sapiens	11	4
8	Methanosarcina mazei	8	8
9	Plautia stali symbiont	2	1
10	Methanoregula formicica	1	29
11	Methanospirillum hungatei	1	12
12	Methanoculleus sp. MAB1	2	
13	Metschnikowia bicuspidata	2	
14	Veillonella parvula	2	
15	Methanothrix soehngenii	1	
16	Coccidioides immitis	1	
17	Xylona heveae	1	
18	Leptosphaeria maculans	1	
19	Colletotrichum orchidophilum	1	
20	Porphyromonas gingivalis	1	
21	Prevotella intermedia	1	
22	Salmonella enterica	1	
23	Haemophilus parainfluenzae	1	
24	Dehalococcoides mccartyi	1	
25	Virgibacillus halodenitrificans	1	
26	Paenibacillus yonginensis	1	
27	Staphylococcus epidermidis	1	
28	Weissella cibaria	1	
29	Clostridium argentinense	1	
30	Oscillibacter valericigenes	1	
31	Veillonella rodentium	1	
32	Methanoregula boonei		19
33	Methanococcus maripaludis		14
34	Methanolacinia petrolearia		5
35	Methanotorris igneus		4
36	Methanothermobacter marburgensis		2
37	Methanoculleus marisnigri		2
38	Methanobacterium paludis		1
39	Methanothermus fervidus		1
40	Methanosarcina lacustris		1
41	Methanolobus psychrophilus		1
42	Candidatus Methanomassiliicoccus intestinalis		1
43	Bacillus subtilis		1
44	Solibacillus silvestris		1
		319	326
